# Shih-Tzu dogs show alterations in ocular surface homeostasis despite adequate aqueous tear production

**DOI:** 10.1186/s13028-024-00724-2

**Published:** 2024-01-17

**Authors:** Rebeca Costa Vitor, Jamille Bispo de Carvalho Teixeira, Katharine Costa dos Santos, Gabriela Mota Sena de Oliveira, Paula Elisa Brandão Guedes, Anaiá da Paixão Sevá, Deusdete Conceição Gomes Junior, Jéssica Fontes Veloso, Renata Santiago Alberto Carlos

**Affiliations:** 1 Department of Agricultural and Environmental Sciences, State University of Santa Cruz (UESC), Ilhéus-Bahia, Brazil; 2 Department of Veterinary Medicine, Federal University of Western Bahia (UFOB), Barra-Bahia, Brazil

**Keywords:** Brachycephalic dogs, Ocular surface, Tear film quality

## Abstract

**Background:**

Shih-Tzu dogs are frequently affected by ocular surface disorders such as corneal ulceration and dry eye disease (DED). The aim of this study was to evaluate ocular surface homeostasis in Shih-Tzu dogs that have adequate aqueous production. Twenty-eight dogs were subjected to eyelid blink counting, Schirmer tear test (STT-1), ophthalmic evaluation, tear film break-up time (TBUT), fluorescein test and Masmali tear ferning (TF) grading scale.

**Results:**

Of the 28 animals evaluated, the median value of incomplete eyelid blinks/min (median = 15.0 blinks/min; Interquartil interval - IQR = 8.7 blinks/min − 19.5 blinks/min) was higher than the complete blinks/min (median = 2.5 blinks/min; IQR = 1.6 blinks/min − 4.3 blinks/min), with statistically significant difference. The Schirmer tear test had a median value of 25.0 mm/min (IQR = 22.7 mm/min − 27.5 mm/min), considered within the normal range for the species. On ophthalmic examination, all dogs had trichiasis of the caruncle and medial lower eyelid entropion. Lagophthalmos was the third most common alteration observed (71.4%; 20/28). The median of TBUT was 4.0 s; (IQR = 3.0 – 6.0 s). All the animals were negative to the fluorescein test and the TFT indicated that the majority of the eyes (51.8%; 29/56) were classified in abnormal grades 3 and 4 according to the Masmali tear ferning (TF) grading scale.

**Conclusions:**

Although the Shith-Tzu dogs had STT-1 values within the normal range for the species there was high prevalence of abnormal TFT grades and low TBUT in all dogs, showing that despite adequate aqueous production, these dogs have poor precorneal tear film quality. In addition, the dogs showed few complete eyelid blinks and ophthalmic alterations, promoting poor tear film diffusion. All these findings, isolated or together, can result in DED.

## Background

The ocular surface is constantly exposed to pathogens and other harmful stimuli, and together with the eyelids, what keeps it protected, moist and nourished is the precorneal tear film (PTF). The PTF is a complex and dynamic trilaminar biofilm composed mainly of lipids, electrolytes, proteins, mucins and water, which provides integrity to the cornea [1–8].

The quality and quantity of tears are fundamental to maintaining a healthy ocular surface, and consequently visual acuity [2, 9]. Variations in tear production and evaporation rates have been observed in different species and breeds, which can be attributed to anatomical, physiological and/or pathological conditions [3, 10].

Given the importance of tear film production and integrity, evaluations of the PTF should be carried out routinely during ophthalmic examinations. The tests that can be carried out include Schirmer tear test (STT-1), which quantitatively assesses the aqueous layer of the tear film; tear film break-up time (TBUT), which assesses the presence or absence of the mucinous layer in the tear film, by reducing the lipid and/or mucin components of the tear film; and staining of the ocular surface with rose bengal/lysamine green and fluorescein, which assess corneal integrity [11, 12]. Other less common tests are conjunctival impression cytology, conjunctival biopsy and tear osmolarity measurement [13, 14].

Studies of the electrolyte composition of tears has revealed that supersaturation of their components forms crystals whose patterns, when dried, can be used for qualitative evaluation [9, 15]. The patterns formed resemble “fern leaves”, and this technique is known as the tear ferning test (TFT) and was first described by Tabbara and Okumoto [15]. In addition to dogs [1], the TFT has also been applied to other species, including horses [16], capuchin monkeys [11] and cats [12].

The most common alteration in the PTF in dogs is dry eye disease (DED), which can occur due to inadequate tear film production or excessive evaporation [17, 18]. In relation to breeds, brachycephalic dogs have anatomical and physiological characteristics that predispose them to development of ocular diseases, such as brachycephalic ocular syndrome [19, 20]. Shih-Tzu dogs are particularly affected by this syndrome, especially those related to PTF [20]. This breed has grown in popularity in recent years and was ranked as the 20th most popular out of 191 breeds in 2020 by the American Kennel Club [21]. Due to the anatomical configuration of its skull, which leads to greater exposure of the eyeball, conditions such as caruncular trichiasis [20], medial lower eyelid entropion [20], exophthalmos [22], lagophthalmos [20, 23], DED [22], and corneal ulcers [17] are frequently reported [19]. As a result of disturbances in tear production or increased tear evaporation, Shih-Tzu dogs can show clinical signs, such as those related to DED, leading to loss of vision [19, 24, 25].

The aim of this study was to assess the quantity and quality of tears in Shih-Tzu dogs using easy-to-perform and cost-effective tests that provide valuable information for veterinarians, including eyelid blink count, STT-1, TBUT and TFT.

## Methods

### Study population and inclusion criteria

The study included 28 dogs (56 eyes): 18 females and 10 males, ranging in age from 1 to 7 years. These dogs were chosen through prior contact with people already known to own purebred Shih-Tzu dogs, without distinction regarding gender or age and without owner´s complaints of ophthalmic disease. The temperature and humidity of the room where the animals were tested were kept between 20.1 and 26.0 °C and 48% and 62% respectively throughout the study.

The inclusion criteria required the animals to be vaccinated against rabies and polyvalent vaccination (distemper, hepatitis, parvovirus, parainfluenza - DHPP) and to show no signs of systemic disease or use of topical or systemic medication. In addition to the clinical assessment to confirm their general state of health, all the animals included in the study had hematological and biochemical tests (including urea, creatinine, alanine aminotransferase and alkaline phosphatase) within normal values for the species [26, 27]. The animals included in the study also had STT-1 > 15 mm/min. All the dogs selected for the study underwent an ophthalmological examination carried out by trained ophtalmologists.

### Evaluation of blinking

The first assessment carried out was the blink count. In order to avoid possible interference in counting due to handling, a five-minute video recording of each animal’s face was made before starting the examination of the ocular surface, for later analysis and counting of complete (when the animal blinks with full coaptation of the eyelids) and incomplete (when the animal does not blink with total closure of the eyelids, leaving a space between them) blink rates, based on the method described in previous studies [7, 28]. The videos were evaluated by three masked examiners (RSAC, JBCT and RSC) at a reduced playback speed (0.5). After counting, the number of blinks was divided by five to obtain the blink count per minute.

### STT-1, tear sampling and TFT

Tear samples were taken between 8:00 and 11:30 a.m., first from the right eye and then from the left eye. For the STT-1, 15.0 mm was considered the minimum reference value for the species [29]. The values were recorded 1 min after inserting the Schirmer tear test strips (Ophthalmos) behind the lower eyelid. As soon as the tear was 30.0 mm wet on the Schirmer strips, which were the same as those used for the STT-1, the strips were immediately placed in a 0.5 mL microtube (Protein LoBind Tubes; Eppendorf, São Paulo, Brazil) and placed in a thermal box until centrifugation. Immediately before centrifugation, the bottom of the 0.5 mL microtube was perforated and it was inserted into a larger 2.0 mL microcentrifuge tube (Protein LoBind Tubes; Eppendorf) to extract the tear fluid, as previously described [12, 16]. The tear fluid was obtained by centrifuging the Schirmer strips (25.8 g for 10 min at 4 °C).

A 2.0 µL tear drop was deposited on a glass slide using a precision pipette, in the center of a circle drawn earlier, and the time taken for the tear to form (i.e., from tear deposition to drying). Total tear drying was observed through visual inspection and it was measured using a digital timer.

After complete drying, the slides were evaluated using a polarized light microscope with 10× magnification and a camera (Microscope Scope A.1/AX10 Axion Cam ICc5; Zeiss, Sao Paulo, Brazil). The images acquired were classified and the formation of branches, angulations and zones of transition were assessed according to the scale of Masmali et al. [9].

The images of the fern patterns were classified by three separate masked examiners (RSAC, JFV and DCGJ) with experience and knowledge in using the scales. The final classification of crystallization patterns was assigned based on the agreement between the classifications of at least two of the three examiners.

### Evaluation of the ocular annexes and intraocular pressure (IOP)

A slit lamp biomicroscope (Vision Class II BL IIIB / YZ30T; Ramos Mejia, São Paulo, Brazil) was used to assess the anterior segment of the eye and ocular appendages. Intraocular Pressure (IOP) was obtained using a rebound tonometer (Tonovet Icare Finland Oy, Vantaa, Finland), with the reference range for dogs being 10.0–26.0 mmHg [30].

### Ocular surface assessment and TBUT

The ocular surface was evaluated with fluorescein dyes (fluorescein test; Ophthalmos), and TBUT was assessed, with a normal value of over 15.0 s [31]. The IOP, fluorescein test and TBUT were carried out after tear sampling to avoid any interference with the TFT. All the data was collected in a room with temperature and humidity controlled. The interval between all the tests was 10.0 min [20].

### Statistical analysis

To assess inter-rater agreement regarding the classification of tear crystals, the Kappa test for ordinal numbers was carried out using the R software with the irr package, and the interpretation was based on the classification described by Lands and Koch [32].The difference between the number of complete and incomplete blinks according to all observers was evaluated using Shapiro-Wilk normality test, and when a nonparametric distribution was established, the paired Friedman test was used, followed by the post-hoc Wilcoxon test with Bonferroni correction for P-value (significance set at *P* < 0.05). These analyses were conducted using the R software (version 3.6.1) with the rstatix package.

Descriptive statistics were computed from the rest of the data.

## Results

### Evaluation of blinking and schirmer tear test

**Fig. 1:**
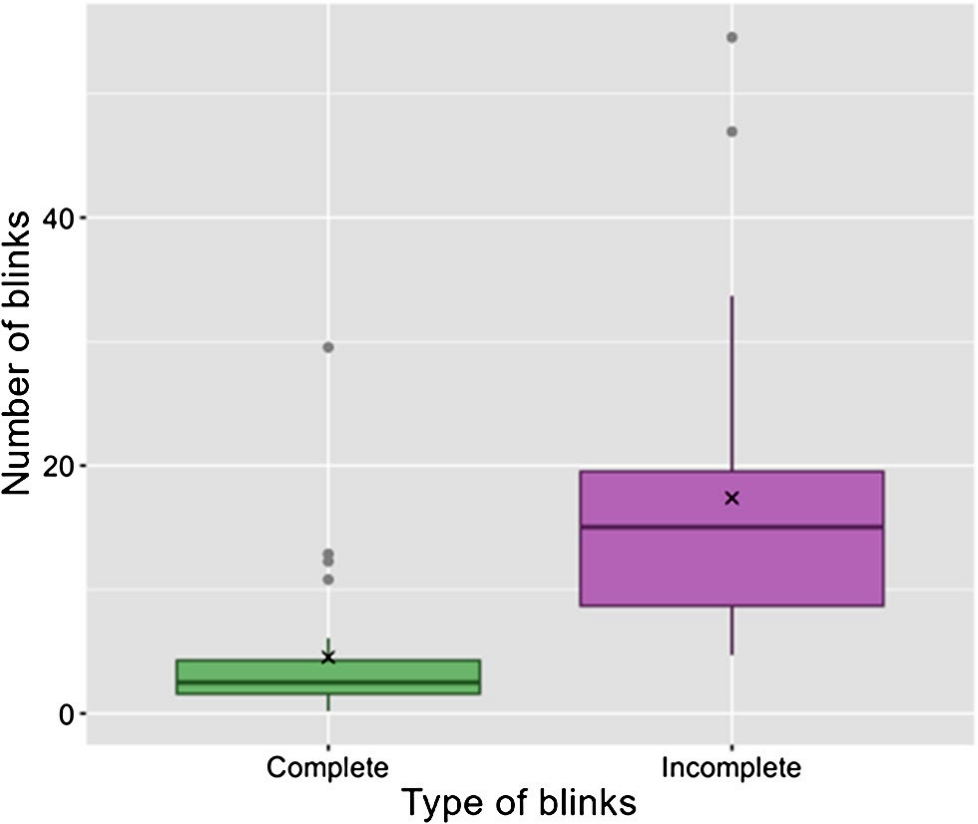
Boxplot of distribution values of complete and incomplete blinks

With regard to counting eyelid blinks, the majority of blinks/min in the 28 dogs with normal STT-1 were considered incomplete by all three observers. Among the blinks observed (total blink rate = 22.0 blinks/min), a minority were considered complete according to all the evaluations by all the observers (W = 693.5; P-value < 0.01). For the complete eyelid blinks/min rate, the median was 2.5 blinks/min (Interval interquartile - IQR = 1.6 blinks/min − 4.3 blinks/min) while for the incomplete eyelid blinks/min rate, the median was 15 blinks/min (IQR = 8.7 blinks/min − 19.5 blinks/min, Fig. 1). According to all the evaluators, most of the eyelid blinks were incomplete (70.9%), with a significant difference (*P* = 0.001) in relation to the number of complete eyelid blinks (20.9%). The median of STT-1 for both eyes was 25.0 mm/min (IQR = 22.7 mm/min − 27.5 mm/min).

### Ophthalmic evaluation

Despite the absence of ophthalmic complaints, all the dogs in the study had medial lower eyelid entropion and trichiasis of the caruncle. Lagophthalmos was the third most common alteration among the dogs, with 71.4% (20/28) of the animals affected. The other alterations are shown in Table 1.

**Table 1:** Ocular alterations found by ophthalmic examination in 28 Shih-Tzu dogs

Alteration	N	%
Caruncle trichiasis	28	100
Medial lower eyelid entropion	28	100
Lagophthalmos	20	71.4
Brown tear stain	16	57.2
Epiphora/excessive secretion	11	39.3
Periocular trichiasis	8	28.6
Corneal fibrosis	7	25.0
Corneal pigmentation	6	21.4
Exophthalmos	5	17.9
Corneal opacity	5	17.9
Pigmentary keratitis	3	10.7

### Tear film break-up time (TBUT) and fluorescein test

The median of TBUT was 4.0 s (IQR = 3.0 − 6.0 s). None of the dogs showed an abnormal fluorescein test pattern.

### Tear ferning test

The median time for the ferning phenomenon to occur in the samples was 18.0 min (IQR = 15.0 − 22.2 min). Based on the ferning analysis of the samples evaluated, the majority of the 56 eyes (51.8%; *n* = 29/56) were classified as grade 3 or 4 (abnormal pattern for dogs) according to the Masmali tear ferning (TF) grading scale [9], as shown in Table 2.

**Table 2:** Classification of lacrimal ferning patterns in the eyes of 28 Shih-Tzu dogs, and their respective percentages, according to the Masmali tear ferning (TF) grading scale [9]

Scale	Classification	Total
**Masmali**	**0**	1 (1.8%)
	**1**	11 (19.6%)
	**2**	15 (26.8%)
	**3**	26 (46.4%)
	**4**	3 (5.4%)

Comparison of the degree of crystallization readings on the Masmali tear ferning (TF) grading scale [9] indicated a strong observer agreement (k = 0.651)

**Fig. 2:**
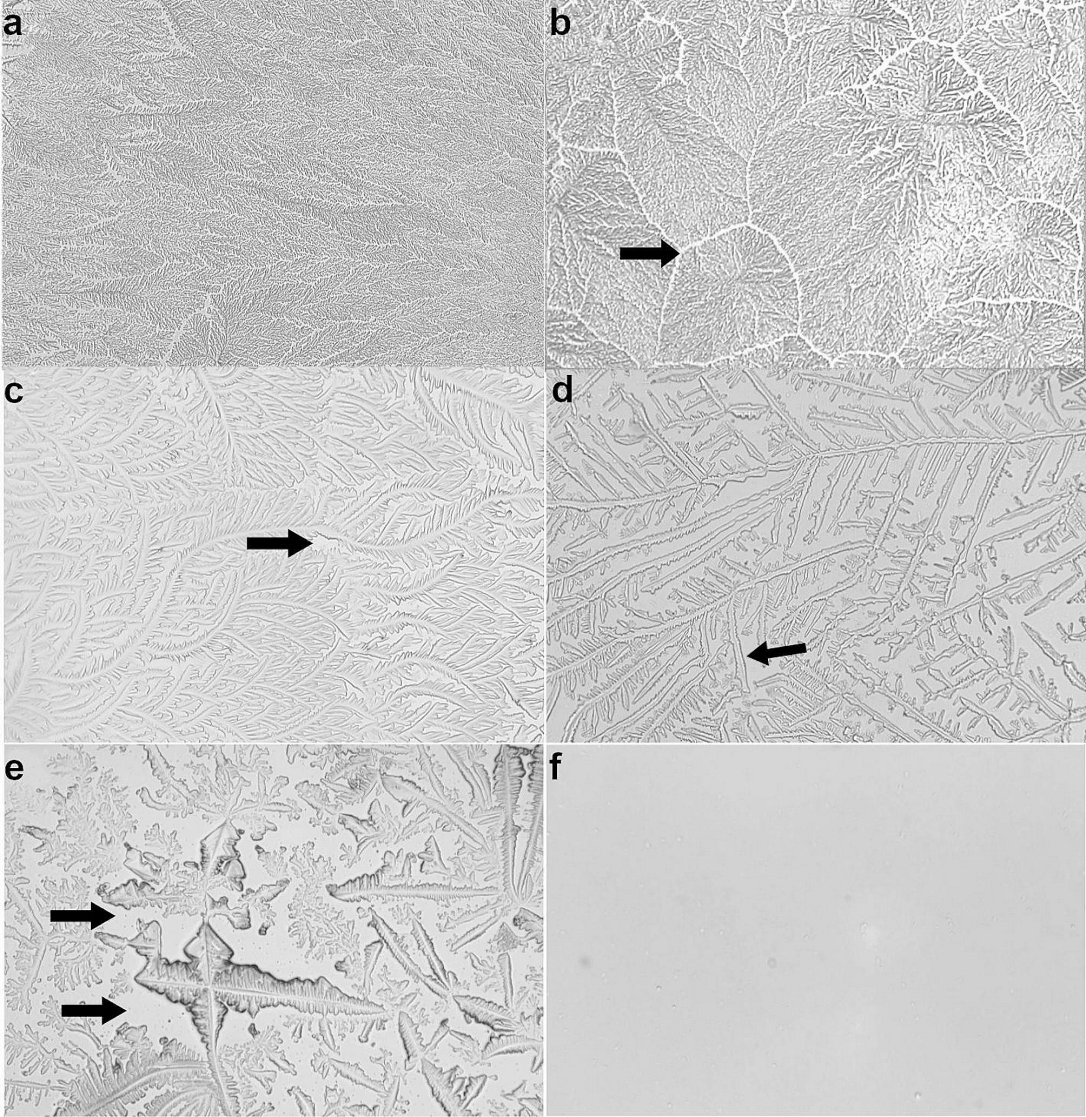
Representation of tear ferning patterns according to the Masmali tear ferning (TF) grading scale [9] in the dogs studied. **(a)** Grade 0: Crystallization with no spaces between the primary branches; **(b)** Grade 1: Slight reduction in the density of the primary branches and the appearance of small spaces between them (arrow); **(c)** Grade 2: The primary and secondary branches are reduced and appear thicker, with greater spacing between them (arrow); and **(d)** Grade 3: The spaces between the primary branches are clearly visible and wide, with the presence of crystals (arrow); **e and f)** Grade 4: There is no presence of well-defined branches, with large spacing between the crystals (arrow) or no crystal formation

The tear ferning patterns classified as Grade 0 and Grade 1 (as described in Table 3), respectively, exhibited high density with dendritic formations showing minimal/null spaces between the primary branches and easily observable nuclei (Fig. 2a-b. In the samples classified as Grade 2 and Grade 3 a loss of definition of the nucleus and large spacing between the primary branches were observed, as well as the formation of coarser crystals (Fig. 2c). The samples classified as Grade 4 had poor tear quality according to the scale, since there was no formation of well-defined branches and there were large spaces between the crystals (Fig. 2e), and even the absence of crystal formation (Fig. 2f).

**Table 3:** Tear Ferning Classification Criteria according to the Masmali tear ferning (TF) grading scale

**Grade 0**	The phenomenon of crystallization is full, with no spaces or gaps between the ferns.
**Grade 1**	The density of the fern and branches is reduced with the appearance of small spaces and gaps between them.
**Grade 2**	The fern and branches are reduced and can become thick and large, with the presence of clear spaces and gaps.
**Grade 3**	The spaces and gaps are increased and very visible in grade 3 without formation of ferns, but with the presence of large crystals.
**Grade 4**	The phenomenon of crystallization in this pattern is totally absent.

## Discussion

Although the STT-1 results were within normal range, our findings corroborate those of Sebbag et al. [20], who also reported an association of Shih-Tzus dogs and tear film deficiencies, showing poor tear film quality in this breed associated with ophthalmic alterations.

Unlike reflex and voluntary blinking, spontaneous blinking is rapid, automatic and unconscious, with complete closure and opening of the eyelids [33, 34]. In order for the tear film to spread over the entire ocular surface, there must be adequate tear production/secretion, as well as appropriate spreading and drainage, to maintain the physiological balance of the cornea [28, 35]. In our study, the dogs evaluated had a high number of incomplete blinks and a low number of complete blinks. This is harmful by favoring rapid tear evaporation, limited spreading and consequent drying of the cornea and conjunctiva, leading to a vicious circle. These alterations can also decrease corneal sensitivity and further reduce blink frequency [8]. Similarly to Kim et al. [36] and Packer et al. [37], we found exophthalmos to be an aggravating factor due to the anatomical characteristics of the Shih-Tzu breed, which has a short snout and a shallow bony orbit, leading to greater exposure of the eyeball and aggravating insufficient eyelid closure [23].

Similar to various studies in Brazil [38] and other countries [36, 39], this study demonstrated the involvement of various ophthalmopathies in Shih-Tzus. As observed by Sebbag et al. [20], 100% of the animals had trichiasis of the caruncle and medial lower eyelid entropion. The third most common condition in this study was lagophthalmos, which was also the third most common alteration found by the mentioned authors. It is thus possible to infer that the high rate of incomplete blinks may also represent the dog’s attempt to compensate the difficulty in performing complete blinks because of these anatomical problems [5].

Although almost half of the dogs evaluated (*n* = 13) had elevated IOP in at least one eye, the diagnosis of glaucoma was not established, since the animals were monitored and showed no clinical signs compatible with this condition. Thus, we attributed this finding to the stress caused by handling, as well as the temperament of the dogs during clinical examinations, which may have temporarily increased IOP in the dogs [40, 41].

The results for TBUT in this study were similar to the values found by Sebbag et al. [20], who also evaluated the ocular surface of 50 Shih-Tzu dogs and found TBUT values of 5.3 s (± 2.4 s). Our findings suggest a possible deficiency of mucin or tear lipids, a situation in which TBUT is generally low, often to less than 5 s [17].

The predominance of grades 3 and 4 in the crystallization pattern in our study, also suggests alterations in PTF even though the STT-1 values were within the reference values for the species as mentioned before. This finding also indicates a qualitative change in the tear film, corroborating Sebbag et al. [20], who stated that although the quantity of tears secreted by Shih-Tzu dogs is often sufficient, their quality may be compromised. The TFT is described as a complementary test to other methods [9, 38, 42, 43]. It is undeniable that the TFT improves understanding of the ocular surface in all species. For this reason, we emphasize that evaluation of tear crystallizations requires training and harmonization between different examiners, as the morphological details of ferning patterns are observer-dependent [12].

## Conclusions

Although the Shih-Tzu dogs had STT-1 values within normal range for the species there was a high prevalence of abnormal TFT pattern and lower TBUT in all dogs, showing that despite adequate aqueous production, these dogs have poor quality of PTF. In addition, the dogs had several ocular surface alterations and few complete eyelid blinks, favoring poor tear film diffusion. All these facts, together or in isolation, can lead Shih-Tzu dogs to develop DED and other conditions such as ulcerative and non-ulcerative keratitis.

## Data Availability

The datasets used and/or analysed during the current study are available from the corresponding author on reasonable request.

## Declarations

### Prior publication

Data have not been published previously.
